# Real-World Applicability of Multiple International Terminology Systems in Oncology: Mapping Study of Clinical Terms for Cancer Diagnosis and Treatment

**DOI:** 10.2196/96548

**Published:** 2026-10-27

**Authors:** Boyun Jang, Eunhye Park, Sangwon Lee, Eunbi Ju, Yewon Jang, In Hae Kwak, Kui Son Choi, Hyun-Joo Kong, Hyo Soung Cha, Hyun-Jin Kim, Heejung Chae

**Affiliations:** 1 Cancer Data Center, National Cancer Control Institute, National Cancer Center, Goyang, South Korea; 2 Department of Medical Information Management, National Cancer Center, Goyang, South Korea; 3 Department of Public Health and Artificial Intelligence, Graduate School of Cancer Science and Policy, National Cancer Center, Goyang, South Korea; 4 Department of Internal Medicine, Seoul National University Bundang Hospital, 82 Gumi-ro 173beon-gil, Seongnam, 13620, South Korea, +82-31-787-7863

**Keywords:** clinical terms, health care terminology, mapping, oncology, Systematized Nomenclature of Medicine Clinical Terms, SNOMED CT, terminology standard

## Abstract

**Background:**

With the rapid expansion of health care data, multiple international standardized terminology systems have been developed and refined to ensure semantic interoperability, which is a crucial factor in facilitating collaborative use.

**Objective:**

This study aimed to evaluate the real-world use and applicability of standardized terminology systems by mapping clinical terms from routine cancer care and to propose guidelines that optimize mapping.

**Methods:**

Clinical terms generated during cancer care at the National Cancer Center Korea were extracted and mapped to multiple target terminology systems: the Korean Standard Classification of Diseases; *International Classification of Diseases, 9th Revision, Clinical Modification*; Logical Observation Identifiers Names and Codes; RxNorm; Anatomical Therapeutic Chemical classification; and Systematized Nomenclature of Medicine Clinical Terms (SNOMED CT). A multidisciplinary working group of health care information managers, data experts, and oncologists verified the mapping outcomes, calculated the cross-verification discordance rate, and compared the coverage across systems.

**Results:**

A total of 3472 source terms were identified across 7 domains: chief complaint, diagnosis, laboratory test, imaging test, surgery/procedure, medication, and radiation therapy. With postcoordination and predefined mapping guidelines, SNOMED CT achieved high overall mapping rates (98.3%‐100%) across domains. Applying postcoordination as needed substantially improved exact semantic match rates, which exceeded 90% in most domains. However, more than 40% of source terms were broadly mapped in the imaging test and radiation therapy domains because procedural details such as imaging technique and radiation field configuration could not be represented in SNOMED CT. The cross-verification discordance rate between mappers, defined as the proportion of source terms with disagreement, remained low (0.3%‐1.3%). RxNorm and Logical Observation Identifiers Names and Codes showed complementary strengths within their specialized domains, adding detail or specificity where SNOMED CT was less complete.

**Conclusions:**

Standardized terminology systems are feasible and effective in encoding clinical terms for cancer care. SNOMED CT was the most comprehensive, and predefined mapping guidelines provide a structured framework for mapping and consensus resolution. Domain-specific systems should be used alongside SNOMED CT, and all systems should be updated regularly to reflect evolving clinical practice.

## Introduction

With advances in IT and precision medicine, health care data volumes have expanded exponentially [1]. The growth of multicenter research and federated analyses has increased the complexity of data exchange. Unlike other big data, health care data originates from diverse sources, many of which are unstructured, creating interoperability challenges when integrating real-world data for clinical research [2]. Effective exchange requires 4 levels of interoperability: foundational, structural, semantic, and organizational. Semantic interoperability, enabling different systems to interpret information with the same meaning, is critical for complex, large-scale health care data [3,4].

Standardized terminology systems that define and organize medical terms have been developed to achieve semantic interoperability. In the 1940s, the World Health Organization (WHO) introduced the *International Classification of Diseases* (*ICD*) for morbidity and mortality statistics, the current version of which is the *ICD-11*. With advances in oncology, more specialized systems have been established, such as the *International Classification of Diseases for Oncology* (*ICD-O*), which codes neoplasms by tumor site (topography) and histology (morphology) [5]. Additional domain-specific systems include the *ICD-10*-Procedure Coding System (*ICD-10*-PCS) for surgical procedures and treatments, Logical Observation Identifiers Names and Codes (LOINC) for laboratory tests, and RxNorm for medications [6-8]. Systematized Nomenclature of Medicine Clinical Terms (SNOMED CT), originating from the College of American Pathologists’ Systematized Nomenclature of Pathology and later merged with the United Kingdom’s Read Codes, is the most widely adopted standardized medical terminology system [9].

Despite substantial progress in terminology development, gaps remain in real-world clinical application. High-quality research requires minimizing information loss during terminology mapping. However, the application of multiple standardized terminology systems to original real-world clinical terms containing detailed, unaltered information has not been widely examined, and potential intermapper discrepancies have not been fully evaluated. Moreover, few studies have addressed the unique complexities of patients with cancer, who undergo more diverse and intricate diagnostic and treatment processes than general patients. In this study, a multidisciplinary team mapped clinical terms from routine cancer care to multiple standardized terminology systems to assess their applicability, identify challenges, and propose guidelines for optimizing real-world clinical terminology mapping.

## Methods

### Working Group

The Cancer Data Center of the National Cancer Center Korea (NCCK), South Korea’s National Cancer Data Center, establishes the data infrastructure and utilization environment required to promote nationwide cancer data research. As part of these efforts, a clinical term-to-standard terminology mapping project was initiated. A multidisciplinary working group of 3 data experts, 7 health information managers, and 2 clinical oncologists was convened. Data experts, who are responsible for building and maintaining the National Cancer Data Center research database and NCCK Clinical Research Data Warehouse (CRDW), conducted source term extraction, integration of the mapping outputs into the database, and quality control to ensure data integrity and operational suitability for standardization and reuse. Five health information managers, with expertise in electronic medical record (EMR) data management, multicenter data standardization, and standard terminology mapping, served as primary mappers. Two senior health information managers (the Chief of Medical Information Management Team and the Terminology Governance Lead of the EMR Steering Committee) supervised the overall mapping process and performed final review and approval of all mapping outputs. The 2 dedicated clinical oncologists, 1 medical oncologist and 1 cancer surgeon, contributed domain expertise in cancer care to review whether the clinical meaning of the source terms was preserved during mapping. When specialized clinical interpretation was required, additional consultation was obtained from relevant physicians within the institution. The working group held regular monthly meetings and biweekly written discussions for project design, progress review, mapping issue discussion, and consensus resolution (Figure 1).

**Figure 1.**
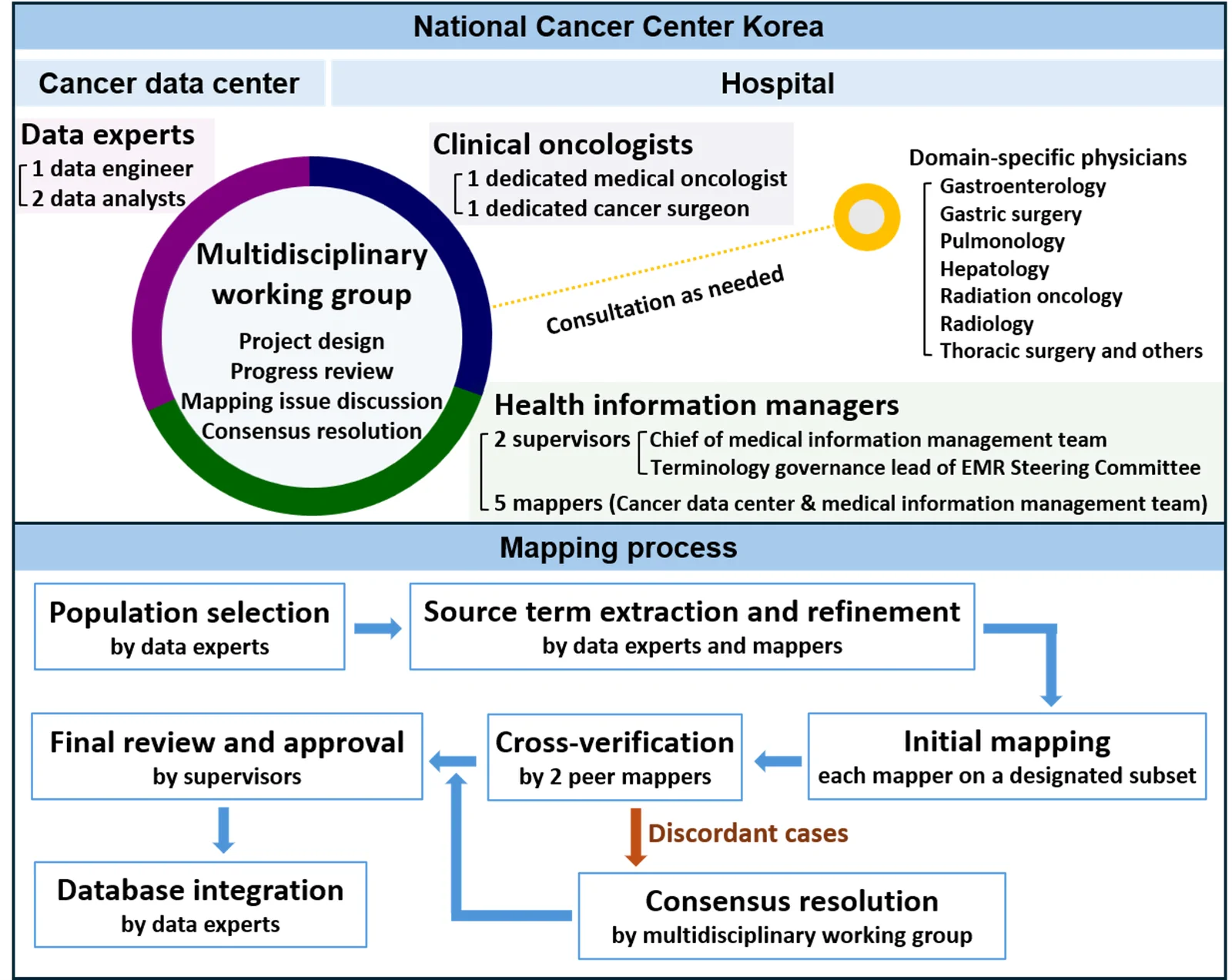
Structure of the multidisciplinary working group and workflow of the standard terminology mapping process. The National Cancer Center Korea established a multidisciplinary working group comprising data experts, certified health information managers, and clinical oncologists, with domain-specific physicians consulted as needed. Each step of the mapping process was carried out by the designated members of the working group, as indicated. EMR: electronic medical record.

### Study Population and Source Terms

Candidate source terms were identified from clinical data in the CRDW, which collects comprehensive information from the EMR system and houses deidentified data for research. Patients diagnosed with one of 6 primary cancers (stomach, breast, liver, colorectal, pancreatic, or lung) at the NCCK between 2010 and 2023 were included. Inclusion was based on *ICD*-O-3 codes: C16 (stomach), C50 (breast), C18/C19/C20 (colorectal), C22 (liver), C25 (pancreas), and C33/C34 (lung). Patients with intrahepatic bile duct cancer (C22.1) and islet cell tumors of the pancreas (C25.4) were excluded. Clinical data were extracted and classified into 7 domains: chief complaint, diagnosis, laboratory test, imaging test, surgery/procedure, medication, and radiation therapy. The source terms underwent minimal refinement to preserve their original clinical meaning: correction of typographical errors and spacing, expansion of abbreviations, removal of duplicates, and splitting of multivalued terms. Electronic data interchange (EDI) reimbursement codes for Korean National Health Insurance (NHI) claims and their effective dates were extracted from the CRDW for reference during mapping.

### Mapping

Each source term was mapped to relevant standardized terminology systems according to its clinical domain. Domain-specific mappings included the Korean Standard Classification of Diseases (KCD), LOINC, *International Classification of Diseases, 9th Revision, Clinical Modification* (*ICD-9-CM*) Volume 3, RxNorm, and the Anatomical Therapeutic Chemical (ATC); all terms, regardless of domain, were mapped to SNOMED CT (Figure 2). Although newer procedure classifications such as *ICD-10*-PCS have been developed, *ICD-9-CM* Volume 3 has been used by many countries and remains in use across several Asian countries because of its established role within national coding infrastructures and the operational complexity of migration [10-14]. In South Korea, *ICD-9-CM* continues to serve as the procedure classification reference for national health statistics, and clinical terms are generated and managed on this basis [15,16]. Therefore, *ICD-9-CM* Volume 3 was selected for the surgery/procedure domain to reflect the source data context.

**Figure 2.**
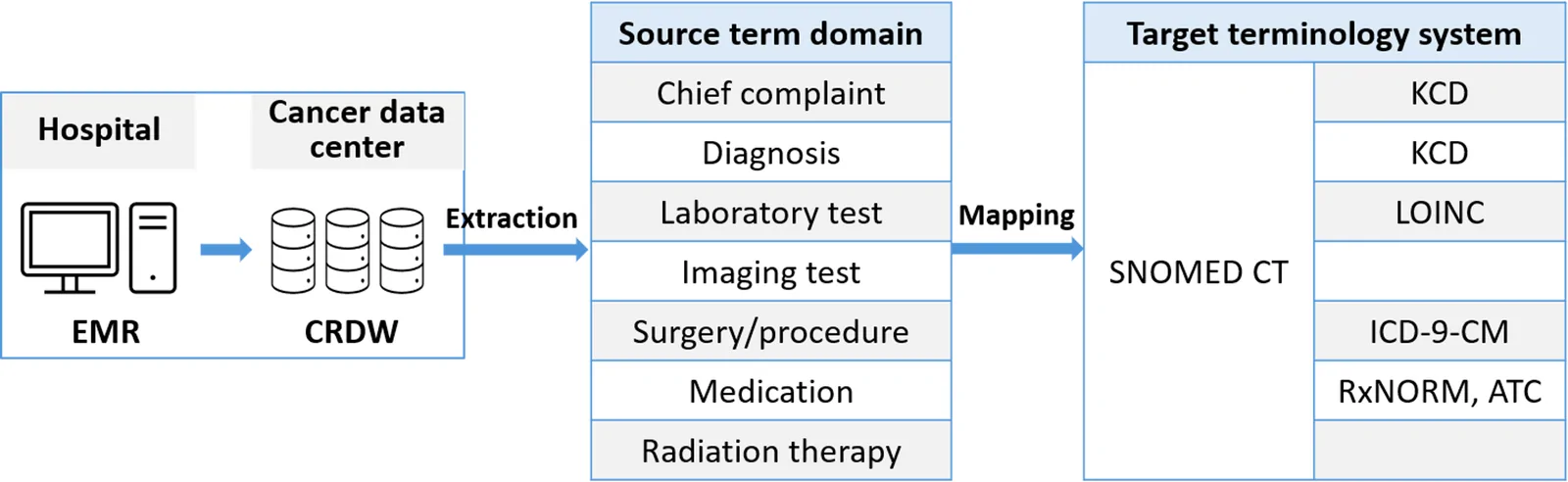
Mapping of clinical source terms to domain-specific standard terminology systems. Clinical terms were extracted from the clinical research data warehouse (CRDW), which contains deidentified data sourced from the hospital’s electronic medical records (EMR) system. Source terms from 7 clinical domains—chief complaint, diagnosis, laboratory test, imaging test, surgery/procedure, medication, and radiation therapy—were mapped to appropriate standardized terminology systems. ATC: Anatomical Therapeutic Chemical; *ICD-9-CM*: *International Classification of Diseases, 9th Revision, Clinical Modification*; KCD: Korean Standard Classification of Diseases; LOINC: Logical Observation Identifiers Names and Codes; RxNorm: normalized naming system for generic and branded drugs.

All mappings followed the commonly recognized rules for each terminology system. For SNOMED CT, mappings adhered to the SNOMED CT Editorial Guide, compositional grammar, and concept model principles [17,18]. For postcoordination, focus concepts, attributes, and attribute values were selected in accordance with the permitted attributes and value ranges defined in the Machine Readable Concept Model (MRCM) [19]. The working group established and shared predefined guidelines with all mappers to specify mapping priorities and ensure consistency when multiple MRCM-compliant mapping options were available within the SNOMED CT framework. SNOMED CT mapping was categorized as “exact” when the target concept matched the source term in meaning, “broad” when the target concept was broader than the source, “narrow” when the target concept was narrower than the source, and “not-mapped” when no suitable target existed. Given the potential loss of semantic specificity and reduced fidelity to the original clinical meaning, narrow mappings were not counted as successful mappings, and grouped with “not-mapped” category as “narrow/not-mapped” in the final mapping performance analysis. Information that the SNOMED CT Editorial Guide identifies as outside the scope of the SNOMED CT International Edition—such as brand names in the medicinal product [20] and administrative or reimbursement-related procedure descriptions, including procedure counts or procedure order [21,22]—was not classified as a broad mapping when absent from the target concept. In contrast, the clinical meaning of the source term itself was considered essential to the mapping and had to be represented in the target concept for the mapping to be classified as exact.

Following initial mapping by 5 mappers each assigned to a designated subset of source terms, 2 peer mappers cross-reviewed each other’s mapping results and recorded agreement or disagreement. Any disagreement was escalated to the multidisciplinary working group for consensus resolution. Clinical oncologists verified that the clinical meaning of each source term was accurately preserved, while data experts and supervising health information managers assessed whether the mapping was operationally appropriate and not overly specific in a way that would compromise standardization and reuse. Final decisions were made by prioritizing (1) accurate preservation of the original clinical meaning and (2) consistent application of predefined mapping principles. Cross-verification results were summarized using the cross-verification discordance rate, defined as the proportion of source terms for which disagreement was identified during cross-verification.

### Ethical Considerations

This study analyzed a terminology dataset of clinical source terms extracted from the institutional deidentified data warehouse and their mapping results, rather than individual-level clinical records. The unit of analysis was the terminology concept rather than the individual patient, and the analytic dataset contained no identifiable personal information or patient-level linkage. Accordingly, this study was granted exemption by the Institutional Review Board of NCCK (No. 2026‐0204), with a waiver of informed consent. The study was conducted in accordance with the principles of the Declaration of Helsinki.

## Results

### Identification of Source Terms

Among the 468,193 patients registered in the CRDW by 2023, 66,380 who were diagnosed with primary stomach, breast, liver, colorectal, pancreatic, or lung cancer between 2010 and 2023 were included. Following extraction, source terms underwent a refinement process, including typographical correction, expansion of abbreviations, duplicate removal, and splitting of multivalued terms (Supplementary Material S1 in Multimedia Appendix 1). No semantic modification or simplification was allowed. A total of 3472 clinical terms were finalized as source terms (Supplementary Material S2 in Multimedia Appendix 1). For the chief complaint domain, the 200 most frequent terms were extracted to prioritize the terms most relevant to routine clinical use. For the medication domain, information on drugs related to cancer prevention and treatment was extracted. All available clinical terms were extracted for the remaining domains.

### Predefined Mapping Guidelines

While all mappings followed the official guidance of each terminology system, the working group established predefined guidelines incorporating oncology-specific considerations to better preserve the original clinical context of source terms and improve consistency across mappers when multiple clinically plausible and terminology-compliant mapping options were available. The predefined guidelines were as follows.

To map chief complaint and diagnosis terms to KCD, if the source term is semantically identical to a subterm of the KCD target term, it should be mapped to that subterm; otherwise, it should be mapped to the main term, which is the primary identifier for the KCD code. In the surgery/procedure domain, if a single-source term contains multiple surgeries or procedures, each surgery or procedure should be mapped individually. The Korean Standard Terminology of Medicine can be used as a supplementary reference for mapping to the *ICD-9-CM* [23]. Although the EDI codes for NHI claims may be considered when available, priority should be given to the meaning of the source term from the CRDW.

For SNOMED CT, each source term should typically be mapped to the relevant hierarchy; however, if a chief complaint indicates the purpose of admission (eg, “for endoscopic variceal ligation”), it should be mapped to the procedure hierarchy. In the medication domain, information outside the scope of SNOMED CT, such as brand name or clinical indication (eg, transcatheter arterial chemoembolization), should be excluded. When the focus concept lacks dose form or strength information, postcoordination should be used. In the radiation therapy domain, if the meaning of a source term is ambiguous, it should be clarified by referencing the EDI codes for NHI claims before mapping. Narrow mappings that might distort the original meaning of the source term should be avoided, and exact mappings should be prioritized over broad mappings. When multiple postcoordinated representations are possible within SNOMED CT Editorial Guide and MRCM-defined attribute constraints, focus concepts, attributes, and attribute values should be selected according to the priority rules summarized in Table 1.

**Table 1.** Systematized Nomenclature of Medicine Clinical Terms (SNOMED CT) postcoordination priorities by clinical domains.

Domain and focus concept	Attribute-value (semantic tag)
Chief complaint	
Symptom	
Select the focus concept corresponding to the main symptom	Postcoordinate “Site (body structure),” if applicable
Reason for admission	
Select “304566005 |Admission for treatment (procedure)|” as the focus concept	Apply postcoordination in the following priority order: ① Treatment name (procedure, regimen/therapy) ② Causative procedure (procedure)
Select “76464004 |Hospital admission, for observation (procedure)|” as the focus concept	Postcoordinate “Test name (procedure),” if applicable
Complications	
Select the focus concept corresponding to the main symptom	Postcoordinate “Causative procedure or surgery (procedure),” if applicable
Diagnosis	
Select the focus concept that best captures the following information, in priority order: ① Behavior + morphology + site ② Morphology + site ③ Behavior + site ④ Behavior + morphology ⑤ Morphology ⑥ Behavior	Postcoordinate additional information not captured by the focus concept in the following priority order: ① Detailed site or laterality (body structure) ② Detailed morphology (morphologic abnormality) ③ Other information (disorder, observable entity, qualifier value, procedure)
Laboratory test	
Select the focus concept that best captures the following information, in priority order: ① Test name + sample type ② Test name	Postcoordinate additional information not captured by the focus concept in the following priority order: ① Test substance (substance, cell) ② (Detailed) sample type (specimen) ③ Test method (procedure) ④ Quantitative/qualitative (qualifier value) ⑤ Other information (qualifier value, physical object, person)
Imaging test	
In-house imaging study	
Select the focus concept that best captures the following information, in priority order: ① Modality + site + position ② Modality + site + injected agents ③ Modality + site ④ Modality + injected agents ⑤ Modality	Postcoordinate additional information not captured by the focus concept in the following priority order: ① (Detailed) site or laterality (body structure) ② Injected agents (substance, procedure) ③ Test method (procedure) ④ Purpose of test (procedure) ⑤ Using device (physical object, physical force) ⑥ Other information (disorder, finding, qualifier value, physical force, person)
Review of outside imaging	
Select “28191001 |Consultation and report by radiologist (procedure)|” as the focus concept	Apply postcoordination in the following priority order: ① Modality + site + injected agents (procedure) ② Modality + site (procedure) ③ (Detailed) site (body structure)
Surgery/procedure	
No additional priority rules	No additional priority rules
Medication	
Select the focus concept that best captures the following information, in priority order: ① Ingredient + dose form + strength ② Ingredient + dose form	Postcoordinate additional information not captured by the focus concept in the following priority order: ① Detailed dose form (dose form) ② Detailed dose form unit (unit of presentation) ③ Numerator value (qualifier value) ④ Numerator unit (qualifier value) ⑤ Denominator value (qualifier value) ⑥ Denominator unit (qualifier value)
Radiation therapy	
No additional priority rules needed	No additional priority rules needed

All mappings followed the SNOMED CT Editorial Guide and MRCM. The priorities listed below were applied additionally, only when multiple valid mapping options existed within these rules.

SNOMED CT concepts are displayed in the format “concept identifier |term|.” The numeric identifier represents the SNOMED CT concept ID, and the term enclosed by vertical bars (“| |”) is the human-readable SNOMED CT term [18]. Numbered items (①, ②, ③, etc) indicate the priority order used in this study for selecting focus concepts or postcoordination attributes when a source term contained multiple mappable elements.

### Mapping Results

Table 2 provides examples of how source terms were mapped to the corresponding target terminology systems in each clinical domain and how the SNOMED CT postcoordination priorities described in Table 1 were applied. Supplementary Material S3 in Multimedia Appendix 1 provides further details on the selection of SNOMED CT postcoordination attributes. For instance, in the case of “Carcinosarcoma, connective and soft tissue, unspecified” in the diagnosis domain, no single target term representing both “① behavior + morphology + site” and “② morphology + site” could be identified. Therefore, the focus concepts “448274000 |Malignant neoplasm of connective tissue (disorder)|” and “269469005 |Malignant neoplasm of soft tissue (disorder)|” were used to capture the “③ behavior (malignant) and the sites (connective and soft tissue)” in a 1:N relationship. The remaining information, morphology, was captured using a postcoordinated expression: “116676008 |Associated morphology(attribute)| = 63264007 |Carcinosarcoma(morphologic abnormality)|.” In the laboratory test domain, following the priority order of “① Test name + sample type,” no single concept capturing both elements was available for “Anti-HCV, immunoassay.” The second priority, test name, was mapped to the focus concept “64411004 | Hepatitis C antibody measurement (procedure)|.” The remaining details were represented through postcoordination by specifying the “② (Detailed) sample type (specimen),” and “③ Test method (procedure)” as follows: “116686009 |Has specimen(attribute)| = 119364003 |Serum specimen (specimen)|” and “370129005 |Measurement method (attribute)| = 104417008 |Immunoassay, qualitative, multiple step method (procedure)|,” respectively.

**Table 2.** Examples of mappings for each domain.

Domain: clinical source terms	Target
Chief complaint: Back pain	
KCD^a^	M54.99 |Dorsalgia, unspecified, site unspecified|
SNOMED CT^b^ (1:1 Exact)	161891005 |Backache (finding)|
Diagnosis: carcinosarcoma, connective and soft tissue, unspecified	
KCD^c^	C49.9 |Malignant neoplasm of connective and soft tissue, unspecified|
SNOMED CT (1:N Exact)	① 448274000 |Malignant neoplasm of connective tissue (disorder)| : { 116676008 |Associated morphology (attribute)| = 63264007 |Carcinosarcoma (morphologic abnormality)| } ② 269469005 |Malignant neoplasm of soft tissue (disorder)| : { 116676008 |Associated morphology (attribute)| = 63264007 |Carcinosarcoma (morphologic abnormality)| }
Laboratory test: Anti-HCV^d^ (test name: hepatitis test and serology; sample name: serum; test unit: X) (EDI^e^: precision immunoassay-hepatitis C antibody)	
LOINC^f^	13955‐0 |Hepatitis C virus Ab [Presence] in Serum or Plasma by Immunoassay|
SNOMED CT (1:1 Exact)	64411004 |Hepatitis C antibody measurement (procedure)| : { 116686009 |Has specimen (attribute)| = 119364003 |Serum specimen (specimen)|, 370129005 |Measurement method (attribute)| = 104417008 |Immunoassay, qualitative, multiple step method (procedure)| }
Imaging test: CT^g^ dynamic liver (contrast)	
SNOMED CT (1:1 Exact)	438591004 |Computed tomography triple phase of liver (procedure)|
Surgery/ procedure: Laparoscopic pelvic lymphadenectomy	
*ICD-9*-*CM*^h^	40.3 | Regional lymph node excision|
SNOMED CT (1:1 Exact)	14059008 |Pelvic lymphadenectomy (procedure)| : { 424226004 |Using device (attribute)| = 86174004 |Laparoscope, device (physical object)| }
Medication: Adriamycin RDF^i^ 10 mg (doxorubicin)	
RxNorm	1790097 |5 ML doxorubicin hydrochloride 2 mg/mL Injection|
ATC^j^	L01DB01 |Doxorubicin|
SNOMED CT (1:1 Exact)	1209144002 |Product containing precisely doxorubicin hydrochloride 2 mg/mL conventional release solution for infusion and/or injection|
Radiation therapy: Intensity modulated radiation therapy [per session] (EDI: IMRT-VMAT)	
SNOMED CT (1:1 Exact)	1156530009 |Volumetric modulated arc therapy (procedure)|

a KCD: Korean Standard Classification of Diseases.

b SNOMED CT: Systematized Nomenclature of Medicine Clinical Terms.

c KCD: Korean Standard Classification of Diseases.

d Hepatitis C virus.

e EDI: EDI codes for NHI claims.

f LOINC: Logical Observation Identifiers Names and Codes.

g CT: computed tomography.

h *ICD-9-CM*: *International Classification of Diseases, 9th Revision, Clinical Modification*.

i RDF: ready to dilute formulation.

j ATC: Anatomical Therapeutic Chemical.

After mapping all the source terms, we examined how each term was represented across multiple target terminology systems to identify system-specific characteristics (Table 3). In the chief complaint and diagnosis domains, KCD could not fully represent certain terms, such as purposes of admission (eg, “for transplantation work-up”) or morphological detail in “adenocarcinoma of sigmoid colon,” whereas SNOMED CT captured these terms via postcoordination. In the surgery/procedure domain, behavioral characteristics (eg, malignancy) could not be included in *ICD-9-CM* procedure codes, whereas SNOMED CT allowed behavior to be represented as attributes in addition to the procedure focus. In the laboratory test domain, LOINC supports the specification of the unit of measure (eg, mg/dL for phosphate) through recommended units, whereas SNOMED CT lacks this level of unit specificity. Conversely, LOINC could not capture additional contextual information, such as “(em)” denoting an emergency procedure, whereas SNOMED CT could via postcoordination. In the medication domain, RxNorm generally represented detailed medication information most completely; for example, it captured ingredient, dose form, strength, and brand name for “Avastin 400 mg injection (bevacizumab).” In contrast, SNOMED CT represented ingredient, dose form, and strength but typically did not include brand name and often expressed strength per unit (eg, 25 mg per 1 mL) rather than total dose. The ATC classification included only ingredient-level information. Although RxNorm was usually the most complete, exceptions existed; for example, for “Mitomycin-C Kyowa,” SNOMED CT provided more complete information than RxNorm.

**Table 3.** Comparison of mapping results according to the target terminology system.

Domain: clinical source terms	Target	
Chief complaint: For transplantation work-up		
KCD^a^	Z04.8 |Examination and observation for other specified reasons|	
SNOMED CT^b^ (1:1 Exact)	76464004 |Hospital admission, for observation (procedure)| : { 363702006 |Has focus (attribute)| = 1303222006 | Pretransplant evaluation of recipient (procedure)| }	
Diagnosis: Adenocarcinoma of sigmoid colon		
KCD^c^	C18.7 |Malignant neoplasm of sigmoid colon|	
SNOMED CT (1:1 Exact)	301756000 |Adenocarcinoma of sigmoid colon (disorder)|	
Laboratory test: Phosphorus (em)		
LOINC^d^	2777‐1 |Phosphate [Mass/volume] in Serum or Plasma|	
SNOMED CT (1:1 Exact)	8364005 |Phosphorus measurement (procedure)| : { 116686009 |Has specimen (attribute)| = 119364003 |Serum specimen (specimen)|, 260870009 |Priority (attribute)| = 25876001 |Emergency (qualifier value)| }	
Surgery/ procedure: Excision of retroperitoneal tumor (malignant)		
*ICD-9-CM*^e^	54.4 |Excision or destruction of peritoneal tissue|	
SNOMED CT (1:1 Exact)	8951004 |Excision of lesion of retroperitoneum (procedure)| : { 363700003 |Direct morphology (attribute)| = 1240414004 |Malignant neoplasm (morphologic abnormality)| }	
Medication: Avastin 400 mg inj (bevacizumab)		
RxNorm	1657074 |16 ML bevacizumab 25 MG/ML Injection [Avastin]|	
ATC^f^	L01FG01 |Bevacizumab|	
SNOMED CT (1:1 Exact)	781788003 |Product containing precisely bevacizumab 25 milligram/1 milliliter conventional release solution for injection (clinical drug)|	
Medication: Mitomycin-c kyowa 10 mg inj (mitomycin)		
RxNorm	1165372 |Mitomycin Injectable Product|	
ATC	L01DC03 |Mitomycin|	
SNOMED CT (1:1 Exact)	326815001 |Product containing precisely mitomycin 10 milligram/1 vial powder for conventional release solution for injection (clinical drug)|	

a KCD: Korean Standard Classification of Diseases.

b SNOMED CT: Systematized Nomenclature of Medicine Clinical Terms.

c KCD: Korean Standard Classification of Diseases.

d LOINC: Logical Observation Identifiers Names and Codes.

e *ICD-9-CM*: *International Classification of Diseases, 9th Revision, Clinical Modification*.

f ATC: Anatomical Therapeutic Chemical.

### Cross-Verification and Consensus Resolution

The initial mapping performed by the assigned mapper was additionally reviewed by 2 peer mappers, who recorded their agreement or disagreement with each mapping result. A mapping was considered discordant if either reviewer disagreed with the initial mapping result, and such cases were referred to the multidisciplinary working group for consensus resolution. There were no major discrepancies in the chief complaint, imaging test, or medication domains; the remaining domains showed cross-verification discordance rates of 0.3% to 1.3%. In the diagnosis domain, the cross-verification discordance rate was 0.3%, primarily involving source terms containing more than 1 anatomical site. This was resolved by adding a rule specifying that if multiple sites in a source term belong to the same parent concept, the term should be mapped to that parent concept rather than mapped 1:N to each child concept. For example, “Neoplasm of uncertain or unknown behavior of liver, gallbladder, and bile ducts” should be mapped to “liver and/or biliary passages,” not separately to “liver,” “gallbladder,” and “bile duct.” The surgery/procedure domain showed a 1.0% cross-verification discordance rate, largely from inconsistent handling of parenthetical details; this was addressed by mapping to target terms that incorporate the parenthetical meaning whenever possible (eg, retaining “malignant” in targets for “excision of retroperitoneal tumor (malignant)”). A 1.3% cross-verification discordance rate in the radiation therapy domain was mostly attributable to limited familiarity with the SNOMED CT relationship groups. Even when the meaning of a source term is not entirely reflected in the fully specified name of a focus concept, relationship group attributes may convey the necessary details, negating additional postcoordination. For example, “external irradiation–high energy radiotherapy” should map to the SNOMED CT concept “high energy beam therapy,” which includes a device attribute in its relationship group that represents external beam delivery, eliminating the need to encode “external irradiation” and “high energy.” Through this process of cross-verification and consensus discussion within the multidisciplinary working group, final mappings were confirmed; as examples, the mapping results for the 20% most frequent terms per each clinical domain are provided in Multimedia Appendix 2.

### Mapping Rates by Standardized Terminology System

Overall mapping rates were high across most domain-system pairs, with the chief complaint domain showing the lowest rates at 97.7% for KCD and 98.3% for SNOMED CT (Supplementary Material S5 in Multimedia Appendix 1). The narrow/not-mapped terms in the chief complaint domain were primarily attributable to ambiguity or insufficient specificity in the source terms, such as “oozing,” “prolapse,” “malfunction,” and “for admission,” for which a semantically appropriate SNOMED CT target could not be reliably selected. In the diagnosis domain, KCD achieved a 100% mapping rate, whereas SNOMED CT achieved 99.9% because a small number of source terms that could only be narrowly mapped (eg, malignant neoplasm of overlapping lesion of other and ill-defined sites) were treated as narrow/not mapped. Outside these two domains, all source terms were successfully mapped to SNOMED CT. In the medication domain, 1.2% of the terms were not mapped to RxNorm owing to the absence of codes for drugs approved only in specific countries (eg, TS-1). All source terms were successfully mapped to other systems (LOINC, *ICD-9-CM* Volume 3, and ATC) within their respective domains.

The detailed SNOMED CT mapping results are presented in Table 4. Among the exact mappings, precoordination predominated in chief complaint (64.7%), diagnosis (79.1%), imaging test (58.9%), surgery/procedure (63.1%), medication (90.1%), and radiation therapy (70.7%), whereas postcoordination was more frequent in laboratory tests (80.2%). In laboratory tests, postcoordination captures additional information such as quantitative/qualitative details and test methods beyond the focus concept. Broad mappings occurred most frequently in the imaging test (43.3%) and radiation therapy domains (40.7%). In the imaging test domain, broad mappings arose mainly because information on view and projection (eg, pelvic inlet and outlet), patient position (eg, decubitus, supine, and erect), and other acquisition techniques (eg, 3D reconstruction and low-dose protocols) could not be represented in the target concepts. In the radiation therapy domain, source terms in the treatment prescription subdomain showed a high broad mapping rate (61.3%), while source terms in the treatment site subdomain achieved a 100% exact mapping rate. Specifically, most broad mappings resulted from incomplete representation of the number of ports (eg, 3 ports) and field arrangement (eg, parallel-opposed 2 fields), whereas information on the preparation processes (eg, simulation and treatment planning), modalities (eg, tomotherapy), and radiation types (eg, proton therapy) was precisely represented in the target concepts.

**Table 4.** Systematized Nomenclature of Medicine Clinical Terms (SNOMED CT) mapping results^a^.

Mapping type^b^	Chief complaint (%)	Diagnosis (%)	Laboratory test (%)	Imaging test (%)	Surgery/procedure (%)	Medication (%)	Radiation therapy (%)^c^
Exact	97.1	97.8	98.3	56.7	89.3	100	59.3
PRE	64.7	79.1	19.8	58.9	63.1	90.1	70.7
POST	34.1	20.2	75.9	39.7	30.6	9.9	29.3
PRE + POST	1.2	0.7	4.3	1.4	6.2	0.0	0.0
Broad^d^	1.2	2.2	1.7	43.3	10.7	0.0	40.7
PRE	0.0	91.2	0.0	89.6	63.8	0.0	83.8
POST	100	8.8	100	10.4	30.4	0.0	16.2
PRE + POST	0.0	0.0	0.0	0.0	5.8	0.0	0.0
Narrow/not-mapped^e^	1.7	0.1	0.0	0.0	0.0	0.0	0.0

a PRE, precoordinated concept (when an existing SNOMED CT concept fully represented the source term); POST, postcoordinated expression (when a single pre-coordinated concept did not fully capture clinically meaningful details).

b Absence of information outside the scope of the SNOMED CT International Edition (eg, brand names, reimbursement-related information) was not classified as a broad mapping.

c By subdomain, mapping results were as follows: Treatment prescription, Exact 38.7% (PRE 32.6%, POST 67.4%), Broad 61.3% (PRE 83.8%, POST 16.2%), and Narrow/not-mapped 0.0%; Treatment site, Exact 100% (PRE 100%, POST 0.0%), Broad 0.0%, and Narrow/not-mapped 0.0%.

d Examples of broad mapping for each clinical domain are provided in Supplementary Material S6 in Multimedia Appendix 1.

e All narrow/not-mapped cases with detailed rationale are summarized in Supplementary Material S7 in Multimedia Appendix 1. These cases were attributable to ambiguity or insufficient specificity of the source terms, which prevented reliable selection of a semantically appropriate SNOMED CT target, rather than to the absence of relevant concepts in SNOMED CT.

## Discussion

We mapped 3472 clinical source terms from real-world records of patients with 6 cancers to multiple standardized terminology systems. Several studies have mapped clinical terms to standardized systems; however, few have documented detailed, real-world mapping experiences. For example, a South Korean study introduced a 9-step process to map local EMR terms to SNOMED CT [4], and a Hungarian group mapped 4661 national procedural codes from 2 university hospitals to SNOMED CT [24]. Although these studies covered a wide range of clinical terms and offered valuable insights, they limited their source terms to specific domains (eg, procedures), restricted their targets to SNOMED CT, and reported ~60% exact mapping rates because postcoordination was not used. In contrast, we focused on patients with cancer, whose care is particularly complex, to better understand the standardization of clinical terms in oncology, where multicenter research demand is high. We included terms across the continuum of cancer care and mapped them not only to SNOMED CT but also to other standardized systems, depending on the clinical domain. We also conducted exhaustive cross-verification for all mappings, whereas previous studies omitted or only partially applied cross-verification.

In our study, SNOMED CT achieved high overall mapping rates, ranging from 98.3% to 100% across all domains, confirming its broad applicability as one of the most comprehensive standardized terminology systems. Applying postcoordination as needed substantially improved exact semantic match rates, which exceeded 90% in most domains. Prior studies have noted lower exact mapping rates in certain domains [25-28]. For instance, Sung [25] et al reported that only 1574/4,111 (38.2%) EDI codes for NHI claims mapped in the laboratory test domain, and Kang et al [28] found a 398/2500 (31.9%) match for therapeutic/surgical procedures; Jung [27] et al showed 11,453/22,610 (50.6%) for medications. However, these rates reflect a reliance on precoordinated concepts. After applying postcoordination, Sung et al [25] and Kang et al [28] improved to 90.4% and 63.6%, respectively. Similarly, our precoordinated match in laboratory tests was 19.8%, rising to 98.3% with postcoordination. SNOMED CT postcoordination also captured details not represented by LOINC attributes, underscoring its role in fully representing the original clinical data and enabling precise use across domains [29,30].

However, even when postcoordination was used, exact semantic match rates remained relatively low in the imaging test and radiation therapy domains, with broad mappings accounting for more than 40% of source terms in each domain. In the imaging test domain, imaging adjustments related to view or projection, patient position, and technique were not permitted in postcoordination, although such information was considered clinically important for the correct acquisition and interpretation of images [31]. Similarly, in the radiation therapy domain, the number of ports and field arrangement could not be represented in SNOMED CT International Edition, despite their relevance to treatment configuration and dosimetric evaluation. Although these limitations reflect the scope and modeling principle of the SNOMED CT International Edition, which does not include certain protocol- or configuration-level details to maintain standardized representation of clinical procedures, our findings suggest that this approach may reduce the semantic fidelity of complex imaging and radiation therapy data that are crucial to cancer care and research. To improve semantic representation, frequently used details could be represented through SNOMED CT national extensions and organized through implementation-specific reference sets. In addition, acquisition parameters and radiation therapy protocol details could be captured using complementary domain-specific terminologies and standards such as Radiology Lexicon and Digital Imaging and Communications in Medicine, including its radiotherapy extension (Digital Imaging and Communications in Medicine – Radiation Therapy) [32-34]. Such a combined approach may help preserve detailed procedural information and improve semantic interoperability in real-world oncology data.

Despite the breadth of SNOMED CT, the complementary use of domain-specific systems is necessary to capture the full characteristics of source terms. For example, although SNOMED CT aligns with the Identification of Medicinal Products, an international standard for dose forms and clinically appropriate strengths, it has limitations with respect to brand names, packaging, and country-specific details (eg, drug codes and reimbursement/insurance status) [35-37]. In contrast, RxNorm represents drug information in greater detail, including multi-ingredient combinations, brand names, exact strengths, and national drug codes. In our study, with precoordination alone, coverage for ingredients, dose form, and strength was same for SNOMED CT (90.1%) and RxNorm (90.1%), but RxNorm more precisely represented the dosage by explicitly encoding the total dose. Although postcoordination can improve SNOMED CT coverage, encoding dosage through postcoordination increases structural complexity, which is a practical limitation. Such country-specific details can also be addressed through a national extension of SNOMED CT, which allows member countries to add concepts and reference sets to meet national requirements while retaining interoperability with the international core [38]. In the United Kingdom, for example, the NHS dictionary of medicines and devices, implemented as the SNOMED CT UK Drug Extension, represents brand names, packaging, and reimbursement-related information using SNOMED CT-compatible identifiers [39]. As South Korea joined SNOMED International in 2020 and is currently developing a national extension, such administrative or policy-related details could also be represented in addition to internationally standardized clinical concepts.

For laboratory tests, LOINC provides a more specialized representation than SNOMED CT via its six-axis model, namely component, property, time, system, scale, and method [40,41]. In our study, precoordinated SNOMED CT concepts alone accounted for only 19.8% of exact SNOMED CT mappings in the laboratory test domain, whereas the remaining 80.2% of exact mappings required postcoordination. Although SNOMED CT postcoordination enabled high mapping rates and captured additional contexts not represented by LOINC (eg, urgent testing status), this limited precoordinated coverage explains the widespread adoption of LOINC-based coding system for laboratory test data in many institutions. These findings support the complementary use of domain-specific terminology systems alongside SNOMED CT. Beyond using these systems in parallel, the recently released LOINC Ontology, a LOINC and SNOMED CT interoperability solution jointly developed by Regenstrief Institute and SNOMED International, may offer a more integrated approach [42,43]. By representing LOINC content within a SNOMED CT concept model, it is expected to enhance LOINC’s computability and improve interoperability between LOINC-coded laboratory observations and broader SNOMED CT-based clinical concepts. Such integrated approaches, rather than the independent use of separate domain-specific terminology systems, may further enhance the semantic interoperability and reusability of real-world clinical data.

To broaden the adoption of SNOMED CT, whose strengths are detailed semantics and wide applicability, the complexity of postcoordination must be addressed [44]. Postcoordination was necessary for 9.9% to 80.2% of the exact mappings in our study. However, SNOMED CT’s flexibility, supporting various precoordinated and postcoordinated expressions, can yield inconsistent code usage across mappers, challenging standardization, because multiple concepts with similar meanings may exist and several valid combinations of focus concepts, attributes, and attribute values may exist [30,45]. In this study, the use of predefined mapping priorities in addition to SNOMED CT Editorial Guide and MRCM supported high consistency in postcoordination decisions (cross-verification discordance rate, 0.3%‐1.3%). To maintain consistency without sacrificing the flexibility of SNOMED CT, it is necessary to incorporate structured mapper training in both clinical domain knowledge and terminology systems, predefined priority rules on selecting focus concepts and attributes, standardized postcoordination templates or reusable expression libraries for recurrent source-term patterns, and cross-verification with regular consensus review. In addition, SNOMED CT content should be periodically updated through the review of frequently used postcoordinated expressions in routine clinical practice, which may be considered as candidates for precoordinated concepts or national extensions.

This study has the limitation in that mappings were developed at a single institution without multicenter validation. Because standard terminology mapping can be influenced by institution-specific workflows, local code management practices, and information system environments, whether the present results can be applied identically to other institutions remains uncertain. Nevertheless, interinstitutional heterogeneity in South Korea might be relatively less pronounced than in health care systems with more fragmented insurance and coding structures. South Korea operates a single-payer NHI system, and medical services are documented and managed using national-level classification systems. In addition, recent government-led initiatives have continued to promote the standardization of health data across institutions, through the establishment of multicenter common clinical data infrastructures (eg, the Data-Driven Hospital Project and the Korea-Clinical Data Utilization Network for Research Excellence), as well as national standards for health data elements terminology (eg, the Korea Core Data for Interoperability) [46,47]. Accordingly, source terms linked to nationally standardized coding systems, such as KCD, LOINC, and EDI claims codes for NHI, are less likely to show large interinstitutional variation and may therefore be more consistently applicable across other institutions. In contrast, locally maintained terms, such as institution-specific names for procedures, surgeries, laboratory tests, and imaging studies, or free-text entries in chief complaints, may yield different mapping results across institutions. Further multicenter validation should focus on these locally maintained terms to determine whether our mapping results remain reproducible across institutions and to identify which source-term categories require local adaptation before broader implementation.

Another limitation concerns the choice of procedure classification system. *ICD-9-CM* Volume 3 was selected for the surgery/procedure domain, considering the Korean clinical coding context in which *ICD-9-CM*–based procedure classification continues to serve as a reference classification for national health statistics [15]. However, this may limit the international applicability of the mapped data. Procedure and intervention classification remains highly heterogeneous across countries. Although the WHO introduced International Classification of Procedures in Medicine in 1978, it was not subsequently maintained, and many countries developed their own procedure classification systems with different structures and purposes, limiting international comparability of intervention data [48-50]. In response to the need for more harmonized frameworks, the WHO has developed the International Classification of Health Interventions to provide a common structure to which codes in national classifications can be mapped for compiling international data [51]. Further studies will be needed to extend the mapping of local clinical terms beyond *ICD-9-CM* toward emerging global standards, thereby enhancing the international interoperability of real-world clinical data.

To the best of our knowledge, this is the first large-scale investigation to compare the strengths and limitations of multiple standardized terminology systems by mapping real-world clinical terms across a full continuum of cancer care. Our study demonstrates the feasibility of applying standardized terminology systems to real-world oncology terms. SNOMED CT is the most comprehensive and universally applicable system, whereas domain-specific systems play essential roles in capturing more specialized aspects of clinical data. Clear mapping priorities in addition to the official guidance for each terminology system, together with cross-verification and consensus review, provided a practical framework for resolving mapping disagreement while maintaining flexibility. Regular updates of standardized terminology systems to accommodate newly emerging clinical terms, together with ongoing mapper education, are important to maintain mapping consistency. Future work may enhance the interoperability of real-world cancer data and contribute to the standardization of research data for the development of clinical decision support systems, health care information exchange, and multicenter research facilitation.

## Supplementary material

10.2196/96548 Multimedia Appendix 1 Supplementary Material S1. Examples of source term refinement prior to mapping Supplementary Material S2. Number of source terms for mapping by clinical domain and cancer type Supplementary Material S3. Examples illustrating the Systematized Nomenclature of Medicine Clinical Terms (SNOMED CT) postcoordination process Supplementary Material S4. Representative mapping results for the most frequent 20% of clinical terms in each domain to standard terminologies Supplementary Material S5. Mapping rates of source terms to each standard terminology system by clinical domain Supplement Material S6. Representative examples of broad mapping in SNOMED CT for each clinical domain Supplementary Material S7. Complete list of not-mapped cases in SNOMED CT mapping and their rationale.

10.2196/96548 Multimedia Appendix 2 Representative mapping results for the most frequent 20% of clinical terms.

## Abbreviations

ATC: Anatomical Therapeutic Chemical
CRDW: Clinical Research Data Warehouse
EDI: electronic data interchange
EMR: electronic medical record
*ICD*: *International Classification of Diseases*
*ICD-10-PCS*: *International Classification of Diseases, 10th Revision, Procedure Coding System*
*ICD-9-CM*: *International Classification of Diseases, 9th Revision, Clinical Modification*
*ICD-O*: *International Classification of Diseases for Oncology*
KCD: Korean Standard Classification of Diseases
LOINC: Logical Observation Identifiers Names and Codes
MRCM: Machine Readable Concept Model
NCCK: National Cancer Center Korea
NHI: National Health Insurance
SNOMED CT: Systematized Nomenclature of Medicine Clinical Terms
WHO: World Health Organization

## References

[1] Matengo W, Otsieno E, Wanjiru K, Chaari L (2020). Digital Health in Focus of Predictive, Preventive and Personalised Medicine.

[2] Amar F, April A, Abran A (2024). Electronic health record and semantic issues using Fast Healthcare Interoperability Resources: systematic mapping review. J Med Internet Res.

[3] (2020). Healthcare Information and Management Systems Society (HIMSS).

[4] Sung S, Park HA, Jung H, Kang H (2023). A SNOMED CT mapping guideline for the local terms used to document clinical findings and procedures in electronic medical records in South Korea: methodological study. JMIR Med Inform.

[5] Fritz AG (2000). International Classification of Diseases for Oncology: ICD-O.

[6] Averill RF, Mullin RL, Steinbeck BA, Goldfield NI, Grant TM (2001). Development of the ICD-10 procedure coding system (ICD-10-PCS). Top Health Inf Manage.

[7] McDonald CJ, Huff SM, Suico JG (2003). LOINC, a universal standard for identifying laboratory observations: a 5-year update. Clin Chem.

[8] Nelson SJ, Zeng K, Kilbourne J, Powell T, Moore R (2011). Normalized names for clinical drugs: RxNorm at 6 years. J Am Med Inform Assoc.

[9] Stearns MQ, Price C, Spackman KA, Wang AY (2001). SNOMED clinical terms: overview of the development process and project status. Proc AMIA Symp.

[10] Zafirah SA, Nur AM, Puteh SEW, Aljunid SM (2018). Potential loss of revenue due to errors in clinical coding during the implementation of the Malaysia diagnosis related group (MY-DRG^®^) Casemix system in a teaching hospital in Malaysia. BMC Health Serv Res.

[11] Opitasari C, Nurwahyuni A (2018). The completeness and accuracy of clinical coding for diagnosis and medical procedure on the INA-CBGs claim amounts at a hospital in South Jakarta. Health Sci J Indones.

[12] Chan KKP, Ng TCC, Sze CY (2026). Validation of diagnosis codes for pleural diseases and procedure codes for relevant respiratory procedures in a healthcare database in Hong Kong: a single tertiary centre study. Hong Kong Med J.

[13] Yu L, Lang J (2020). Diagnosis-related Groups (DRG) pricing and payment policy in China: where are we?. Hepatobiliary Surg Nutr.

[14] Giordani B, Burgio A, Grippo F, Barone A, Eugeni E, Baglio G (2024). The use of ICD-9-CM coding to identify COVID-19 diagnoses and determine risk factors for 30-day death rate in hospitalized patients in Italy: retrospective study. JMIR Public Health Surveill.

[15] Korean Health Information Management Association (2023). Study on development of Korean classification of health interventions, year 7 [Report in Korean]. https://mlib1.kostat.go.kr/search/detail/CATSUZ000000099128?location=A0000001.

[16] Lee YK, Hong SO, Park SJ (2021). Data resource profile: the Korea National Hospital Discharge In-depth Injury Survey. Epidemiol Health.

[17] (2025). SNOMED CT editorial guide. SNOMED International.

[18] (2025). Compositional grammar specification. SNOMED International.

[19] (2026). SNOMED CT Machine Readable Concept Model. SNOMED International.

[20] Medicinal product. SNOMED International.

[21] (2025). Out of scope. SNOMED International.

[22] (2026). Procedure modeling. SNOMED International.

[23] Korean Standard Terminology of Medicine (KOSTOM), version 7. https://www.hins.or.kr.

[24] Mészáros Á, Kovács S, Héja T, Bagyura Z, Zemplényi A (2023). Mapping Hungarian procedure codes to SNOMED CT. BMC Med Res Methodol.

[25] Sung S, Park HA, Park SK, Jung H, Kang H, Lee MS (2023). Mapping Korean National Health Insurance claim codes for laboratory test to SNOMED CT. Stud Health Technol Inform.

[26] Hwang EJ, Park HA, Sohn SK (2019). Mapping Korean EDI medical procedure code to SNOMED CT. Stud Health Technol Inform.

[27] Jung H, Park HA, Kang H, Lee M, Sung S, Park S (2022). Mapping Korean National Health Insurance pharmaceutical claim codes to SNOMED CT. Stud Health Technol Inform.

[28] Kang H, Park HA (2022). Mapping Korean National Health Insurance reimbursement claim codes for therapeutic and surgical procedures to SNOMED-CT to facilitate data reuse. Stud Health Technol Inform.

[29] Richesson RL, Andrews JE, Krischer JP (2006). Use of SNOMED CT to represent clinical research data: a semantic characterization of data items on case report forms in vasculitis research. J Am Med Inform Assoc.

[30] Campbell JR, Xu J, Fung KW (2011). Can SNOMED CT fulfill the vision of a compositional terminology? Analyzing the use case for problem list. AMIA Annu Symp Proc.

[31] (2025). Imaging adjustments for view, projection, or technique. SNOMED International.

[32] Marquis M, Bossenko I, Ross P (2025). RadLex and SNOMED CT integration: a pilot study for standardising radiology classification. Insights Imaging.

[33] Kahn CE, Langlotz CP, Channin DS, Rubin DL (2011). Informatics in radiology: an information model of the DICOM standard. Radiographics.

[34] Law MYY, Liu B (2009). Informatics in radiology: DICOM-RT and its utilization in radiation therapy. Radiographics.

[35] Bodenreider O, Cornet R, Vreeman DJ (2018). Recent developments in clinical terminologies—SNOMED CT, LOINC, and RxNorm. Yearb Med Inform.

[36] Bodenreider O, James J (2018). The new SNOMED CT International Medicinal Product model. CEUR Workshop Proc.

[37] Noel Nikiema J, Bodenreider O (2019). Comparing the representation of medicinal products in RxNorm and SNOMED CT—consequences on interoperability. CEUR Workshop Proc.

[38] (2025). Extension and customization. SNOMED International.

[39] Dictionary of medicines and devices (dm+d). NHS England Digital.

[40] LOINC users’ guide. LOINC.

[41] Bodenreider O (2008). Issues in mapping LOINC laboratory tests to SNOMED CT. AMIA Annu Symp Proc.

[42] (2026). Implementation guide for the LOINC ontology. SNOMED International.

[43] Regenstrief Institute & SNOMED International.

[44] Benson T (2012). Principles of Health Interoperability HL7 and SNOMED.

[45] Miñarro-Giménez JA, Martínez-Costa C, Karlsson D, Schulz S, Gøeg KR (2018). Qualitative analysis of manual annotations of clinical text with SNOMED CT. PLoS One.

[46] Lee S, Choi YH, Kim HM (2025). The Cancer Clinical Library Database (CCLD) from the Korea-Clinical Data Utilization Network for Research Excellence (K-CURE) project. Cancer Res Treat.

[47] Kwon A, Lee HY, Shin SY (2024). Current health data standardization project and future directions to ensure interoperability in Korea. Healthc Inform Res.

[48] (1978). International Classification of Procedures in Medicine.

[49] Paviot BT, Madden R, Moskal L (2011). Development of a new international classification of health interventions based on an ontology framework. Stud Health Technol Inform.

[50] Mellinghoff SC, Bruns C, Al-Monajjed R (2022). Harmonized procedure coding system for surgical procedures and analysis of surgical site infections (SSI) of five European countries. BMC Med Res Methodol.

[51] WHO-FIC maintenance platform. International Classification of Health Interventions (ICHI).

