# Supplementary material for: Real-World Applicability of Multiple International Terminology Systems in Oncology: Mapping Study of Clinical Terms for Cancer Diagnosis and Treatment
Source: JMIR Med Inform. 2026 Oct 27;14:e96548. doi: 10.2196/96548 (PMC13712170; doi:10.2196/96548)
Supplement: Supplementary file 1 [file medinform-v14-e96548-s001.docx]

**Supplementary Material S1. Examples of source term refinement prior to mapping**

(1) Correction of typographical errors and spacing

| Original source term | Refined source term |
| --- | --- |
| Malignat Neoplasm Of Skin | Malignant neoplasm of skin |
| curative resection of esophageal malignant tumor-thoracicand abdominal approach | Curative resection of esophageal malignant tumor – thoracic and abdominal approach |

Red text indicates the portions of source terms modified during refinement.

(2) Expansion of abbreviations

| Original source term | Refined source term |
| --- | --- |
| Mammo CC & MLO (Both) | Mammography CC & MLO (Both) |

Red text indicates the portions of source terms modified during refinement.

(3) Removal of duplicate terms

| Original source term | Refined source term |
| --- | --- |
| Abdominal pain | Abdominal pain |
| Abd pain |  |
| Abd.pain |  |
| Abdomen pain |  |

(4) Splitting of multi-valued terms

| Original source term | Refined source term |
| --- | --- |
| Fever, diarrhea | Fever |
|  | Diarrhea |

**Supplementary Material S2. Number of source terms for mapping by clinical domain and cancer type**

| Domain | | Stomach cancer | Breast cancer | Liver cancer | Colorectal cancer | Pancreatic cancer | Lung cancer | Total | Total minus duplicates |
| --- | --- | --- | --- | --- | --- | --- | --- | --- | --- |
| Chief complaint | | 62 | 64 | 72 | 70 | 75 | 68 | 411 | 172 |
| Diagnosis | | 728 | 690 | 387 | 751 | 288 | 804 | 3,648 | 1,563 |
| Laboratory test | | 23 | 59 | 80 | 66 | 66 | 14 | 308 | 118 |
| Imaging test | | 438 | 148 | 168 | 107 | 146 | 42 | 1,049 | 644 |
| Surgery/procedure | | 331 | 123 | 56 | 143 | 14 | 118 | 785 | 647 |
| Medication | | 72 | 94 | 32 | 98 | 37 | 48 | 381 | 161 |
| Radiation therapy | Treatment site | 36 | 26 | 25 | 39 | 23 | 48 | 197 | 56 |
|  | Treatment prescription | 71 | 50 | 74 | 77 | 28 | 102 | 402 | 111 |
| Total | | 1,761 | 1,254 | 894 | 1,351 | 677 | 1,244 | 7,181 | 3,472 |

**Supplementary Material S3. Examples illustrating the SNOMED CT post-coordination process**

| Source term | Carcinosarcoma, connective and soft tissue, unspecified |
| --- | --- |
| Target term | ① 448274000 \|Malignant neoplasm of connective tissue (disorder)\| : { 116676008 \|Associated morphology (attribute)\| = 63264007 \|Carcinosarcoma (morphologic abnormality)\| }  ② 269469005 \|Malignant neoplasm of soft tissue (disorder)\| : { 116676008 \|Associated morphology (attribute)\| = 63264007 \|Carcinosarcoma (morphologic abnormality)\| } |

| Source term | Anti-HCV (Hepatitis test and serology; serum)  (EDI for NHI claims: Precision Immunoassay - Hepatitis C antibody) |
| --- | --- |
| Target term | 64411004 \|Hepatitis C antibody measurement (procedure)\| :  { 116686009 \|Has specimen (attribute)\| = 119364003 \|Serum specimen (specimen)\|,  370129005 \|Measurement method (attribute)\| = 104417008 \|Immunoassay, qualitative, multiple step method (procedure)\| } |

| Target term | 14059008 \| Pelvic lymphadenectomy (procedure) \| :  { 424226004 \|Using device (attribute)\| = 86174004 \|Laparoscope, device (physical object)\| } |
| --- | --- |

**Supplementary Material S4. Representative mapping results for the most frequent 20% of clinical terms in each domain to standard terminologies**

Supplementary Material S4 is provided as a separate Excel file.

**Supplementary Material S5. Mapping rates of source terms to each standard terminology system by clinical domain**

| Domain | | KCD | ICD-9-CM | LOINC | ATC | RxNorm | SNOMED CT |
| --- | --- | --- | --- | --- | --- | --- | --- |
| Chief complaint | | 97.7% |  |  |  |  | 98.3% |
| Diagnosis | | 100% |  |  |  |  | 99.9% |
| Laboratory test | |  |  | 100% |  |  | 100% |
| Imaging test | |  |  |  |  |  | 100% |
| Surgery/procedure | |  | 100% |  |  |  | 100% |
| Medication | |  |  |  | 100% | 98.8% | 100% |
| Radiation therapy | Treatment site |  |  |  |  |  | 100% |
|  | Treatment prescription |  |  |  |  |  | 100% |

**Supplementary Material S6. Representative examples of broad mapping in SNOMED CT for each clinical domain**

| Domain | Source term | Target Term | Reason for broad mapping |
| --- | --- | --- | --- |
| Chief complaint | PTBD drain decrease | 304566005 \|Admission for treatment (procedure)\| :  { 363702006 \|Has focus (attribute)\| = 385946001 \|Drainage tube care management (procedure)\| } | The change in drainage output ("decrease") could not be encoded, as no pre-coordinated concept represented it in this context and the procedure-domain concept model does not permit a post-coordination attribute to specify a quantitative change; this resulted in a broad mapping. |
| Diagnosis | Conventional chondrosarcoma, bone of limb | 723843005 \|Primary chondrosarcoma of bone of limb (disorder)\| | The histological subtype ("conventional") could not be encoded, as no pre-coordinated concept was available for this subtype and no morphology value permitted under the concept model corresponded to it; this resulted in a broad mapping. |
| Laboratory test | Prothrombin time (Sec), Citrate blood | 396451008 \|Prothrombin time (procedure)\| :  { 116686009 \|Has specimen (attribute)\| = 1351816006 \|Whole blood specimen with citrate (specimen)\| } | The reporting unit ("sec") could not be encoded, as the procedure-domain concept model does not permit a post-coordination attribute to specify units of measurement; this resulted in a broad mapping. |
| Imaging test | CT Abdomen & Pelvis LOW DOSE (Contrast) | 432370003 \|Computed tomography of abdomen and pelvis with contrast (procedure)\| | The acquisition protocol ("low-dose") could not be encoded, as no pre-coordinated concept represented low-dose contrast-enhanced CT of the abdomen and pelvis and the concept model does not permit a post-coordination attribute to specify radiation dose; this resulted in a broad mapping. |
| Surgery/ procedure | Radical Cholecystectomy of Gallbladder Cancer | 38102005 \|Cholecystectomy (procedure)\| :  { 363702006 \|Has focus (attribute)\| = 363353009 \|Malignant neoplasm of gallbladder (disorder)\| } | The extent of resection ("radical") could not be encoded, as no pre-coordinated concept represented radical cholecystectomy and the concept model does not permit a post-coordination attribute to specify the extent of resection; this resulted in a broad mapping. |
| Medication | No broad mapping occurred in this domain | | |
| Radiation therapy | Intensity-Modulated Radiation Therapy – Static (Insurance Covered) | 441799006 \|Intensity modulated radiation therapy (procedure)\| | The beam delivery technique ("static") could not be encoded, as no pre-coordinated concept represented static IMRT and the concept model does not permit a post-coordination attribute to specify the delivery technique; this resulted in a broad mapping. |

**Supplementary Material S7. Complete list of narrow/not-mapped cases in SNOMED CT mapping and their rationale**

| Source Term | Candidate target concepts | Reason for final not-mapped classification |
| --- | --- | --- |
| Oozing | • Cord oozing  • Wound oozing  • Oozing of pigmented skin lesion | Insufficient information on the anatomical site or type of discharge; mapping to a specific SNOMED CT candidate would introduce semantic distortion or unintended semantic augmentation. |
| Malfunction | • Stoma malfunction  • Ileostomy malfunction  • Equipment malfunction  • Colostomy malfunction  • Cystostomy malfunction  • Gastrostomy malfunction  • Enterostomy malfunction  • Jejunostomy malfunction  • Esophagostomy malfunction  • Malfunction of tracheostomy  • Malfunction of insulin pump | Insufficient information on the device or system involved; mapping to a specific SNOMED CT candidate would introduce semantic distortion or unintended semantic augmentation. |
| Prolapse | • Anal prolapse • Cord prolapse  • Iris prolapse • Uveal prolapse  • Prolapsed pile • Stomal prolapse  • Rectal prolapse • Gastric prolapse  • Uterine prolapse • Genital prolapse  • Duodenal prolapse • Vitreous prolapse  • Prolapse of ovary • Colostomy prolapse | Insufficient information on the anatomical site or organ involved; mapping to a specific SNOMED CT candidate would introduce semantic distortion or unintended semantic augmentation. |
| Malignant neoplasm of overlapping lesion of other and ill-defined sites | • Malignant neoplasm, overlapping lesion of penis  • Malignant neoplasm, overlapping lesion of breast  • Malignant neoplasm, overlapping lesion of larynx  • Malignant neoplasm, overlapping lesion of stomach  • Malignant neoplasm, overlapping lesion of bladder  • Malignant neoplasm, overlapping lesion of pancreas  • Malignant neoplasm, overlapping lesion of esophagus  • Malignant neoplasm, overlapping lesion of hypopharynx  • Malignant neoplasm, overlapping lesion of nasopharynx  • Malignant neoplasm, overlapping lesion of biliary tract | Insufficient information on the involved overlapping site; mapping to a specific SNOMED CT candidate would introduce semantic distortion or unintended semantic augmentation. |
